# Machine-Learning-Based Noninvasive In Vivo Estimation of HbA1c Using Photoplethysmography Signals

**DOI:** 10.3390/s22082963

**Published:** 2022-04-12

**Authors:** Tae-Ho Kwon, Ki-Doo Kim

**Affiliations:** School of Electrical Engineering, Kookmin University, Seoul 02707, Korea; kmjkth@kookmin.ac.kr

**Keywords:** photoplethysmography, HbA1c, diabetes, features, machine learning

## Abstract

Glycated hemoglobin (HbA1c) is an important factor in monitoring diabetes. Since the glycated hemoglobin value reflects the average blood glucose level over 3 months, it is not affected by exercise or food intake immediately prior to measurement. Thus, it is used as the most basic measure of evaluating blood-glucose control over a certain period and predicting the occurrence of long-term complications due to diabetes. However, as the existing measurement methods are invasive, there is a burden on the measurement subject who has to endure increased blood gathering and exposure to the risk of secondary infections. To overcome this problem, we propose a machine-learning-based noninvasive estimation method in this study using photoplethysmography (PPG) signals. First, the development of the device used to acquire the PPG signals is described in detail. Thereafter, discriminative and effective features are extracted from the acquired PPG signals using the device, and a machine-learning algorithm is used to estimate the glycated hemoglobin value from the extracted features. Finally, the performance of the proposed method is evaluated by comparison with existing model-based methods.

## 1. Introduction

The blood in our body is composed of 55% yellow liquid plasma, 44% solid red blood cells, and 1% white blood cells and platelets. Hemoglobin is a type of protein found in the red blood cells and plays a major role in transporting oxygen by binding to oxygen in the blood and also binding to the glucose present in the blood cells. Hemoglobin bound to glucose is called glycated hemoglobin (HbA1c); with respect to glycated hemoglobin, when there is more glucose in the blood, greater amounts of hemoglobin and glucose in the red blood cells combine to reflect the increase in blood glucose levels. The glycated hemoglobin levels refer to the proportion of glycated hemoglobin in the blood relative to the total amount of hemoglobin. Although the lifespan of a red blood cell is about 120 days, the lifespan of a glycated red blood cell is slightly shorter such that the glycated hemoglobin level reflects the average blood glucose level over 3 months. In general, instantaneous blood glucose levels can change significantly because of food or environment, such as diet, smoking, coffee consumption, and exercise, and are less reliable or may be inaccurate during testing. On the other hand, as glycated hemoglobin is not affected by immediate activities or food intake, it is used as the most basic measure of evaluating blood-glucose control over a certain period of time and to predict the occurrence of long-term complications due to diabetes [1].

Conventional methods for measuring HbA1c are mostly invasive, which increases the blood collection burden on the measurement subject and may cause skin and red blood cell lifespan abnormalities; it may also cause problems with inaccurate measurements in case of pregnancy or kidney disease [2,3,4,5,6]. In this study, to overcome this problem, we propose a machine-learning-based noninvasive estimation method using photoplethysmography (PPG) signals.

PPG is an optically acquired plethysmogram that can be used to detect changes in blood volume in the microvascular tissue layer. The blood volume change can be identified as the peak or valley of the PPG signal. As the blood flow in the dermis can be altered by several physiological systems, PPG can also be used to monitor respiration, hypovolemia, and other circulatory conditions. In addition, the shape of the PPG waveform may differ between subjects and depends on the location, where the pulse oximeter (PO) is attached, and the method (transmission or reflection). 

The body part of the subject to be measured may include a portion capable of detecting capillaries existing under the skin according to the thickness of the skin. The light wave received by the photodetector (PD) is usually called the PPG signal, or the digital volume pulse (DVP). The PPG signal uses a light-emitting diode (LED) as the transmitter and the PD as the receiver, and the glycated hemoglobin level can be calculated on the basis of the ratio of the AC to DC parts of the PPG signals. Our previous studies on model-based methods for estimating glycated hemoglobin were presented in [7,8,9]. However, since these methods use a simplified body model through mathematical modeling, there are differences from an actual body. Therefore, calibration is needed to overcome the differences between the model and actual results. In the present study, we propose a method that uses machine learning (ML) to estimate the glycated hemoglobin from PPG signals and derives results without separate mathematical modeling.

## 2. Related Research

In general, there are several available methods to measure blood-glucose levels. At home, self-blood glucose monitoring (SBGM) mainly involves blood glucose measurement from blood collected from the capillaries at the fingertips; however, at medical institutions, the blood glucose levels in the plasma are determined by separating it from the blood collected from a vein. Moreover, in recent years, continuous blood glucose monitoring (CBGM) methods are used often, where measurements are obtained from the interstitial fluid via attachment to the skin of the abdomen or forearm through a patch that administers insulin frequently to patients who have diabetes mellitus. However, these methods are invasive and may cause burden and problems with blood collection.

**Figure 1:**
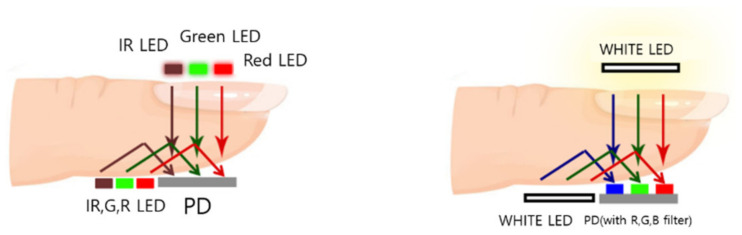
Example of LED–PD arrangement for HbA1c measurement.

We previously studied a noninvasive method for estimating glycated hemoglobin through PPG signals [7,8,9]; these signals were acquired using three LEDs (each providing light of a different wavelength) at the transmitting end with the PD at the receiving end. Both HbA1c and blood oxygenation level (SpO_2_) are parameters that depend on the blood components and both parameters are estimated on the basis of the ratio of the AC to DC parts of the PPG signals. Light is irradiated from three LEDs with different wavelengths to different parts of the body (finger, wrist, forehead, cheek, ear, etc.), and the transmitted and reflected light waves are detected by an optical PD. Figure 1 shows an example of the LED–PD placement for glycated hemoglobin estimation.

In our previous study [7,8,9], three light sources were used to simultaneously estimate two unknown variables (%HbA1c and %SpO_2_). These light sources are used to design an optical system based on the model of either the Beer–Lambert law or photon-diffusion theory. Both HbA1c and SpO_2_ concentrations can be estimated from each model, which has distinct advantages and disadvantages. The PPG signals can be simply and mathematically modeled as a homogeneous mixture of skin tissue, arterial and venous blood, and water at the location of signal acquisition (e.g., finger, wrist, earlobe). Bone is generally ignored because bone tissue is assumed to fully absorb the light, so it does not transmit or reflect/scatter enough light to be detected by the PD. The existing model treats HbA1c, HbO (oxyhemoglobin), and HHb (de-oxyhemoglobin) as the three major components of blood. The detailed descriptions of these models can be found elsewhere [7,8].

In addition, we studied a method of estimating blood glucose levels prior to glycated hemoglobin estimation using ML [10]; in [10], we first extracted features from PPG signals and then used an ML model to estimate the blood glucose level. In the present study, we use ML to estimate the glycated hemoglobin values. However, unlike the previous study [10], we present performance improvement results based on importance-based feature selection and optimal multi-wavelength selection.

Based on our previous studies, the goals and directions for the present study are established as follows:

- Development of glycated hemoglobin estimation process based on ML;
- Selection of optimal wavelength combinations and features for glycated hemoglobin estimation;
- Diabetes status classification through glycated hemoglobin estimation.

## 3. Proposed Method

Machine learning can help construct regression and classification models for HbA1c estimation. The glycated hemoglobin level learning algorithm proposed herein uses two ensemble method-based models—random forest (RF) and extreme gradient boosting (XGB)—for estimating the glycated hemoglobin value.

### 3.1. PPG Signal Processing

**Figure 2:**
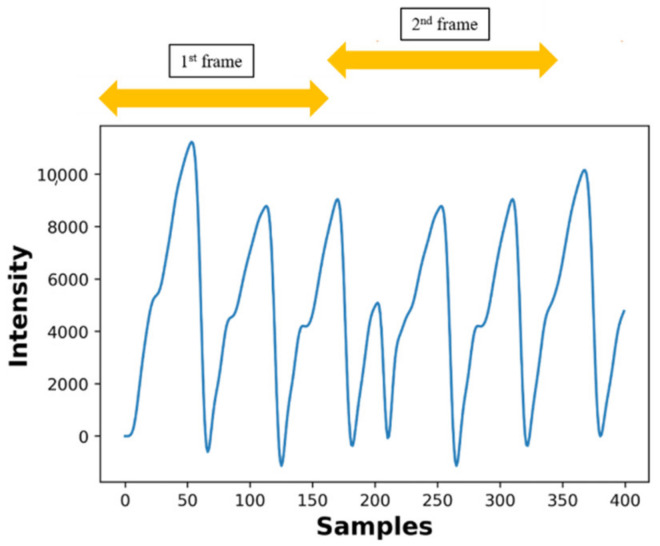
Signal segmentation for 3 s after preprocessing [10].

In the preprocessing step, the PPG signals were filtered through several stages, and the high- and low-frequency noises were removed by filtering. A sixth-order Butterworth lowpass filter with a −3 dB point at 8 Hz was used to remove the high-frequency noise. In addition, as shown in Figure 2, the PPG signal was subdivided into 3 s intervals at a sampling rate of 56 samples per second, which resulted in about 168 samples over the 3 s interval. Thereafter, the 3 s PPG signal was passed through the feature extraction module.

### 3.2. Feature Extraction

In the feature extraction step, 15 important and distinct features were extracted from the PPG signal. First, the important features were selected on the basis of our previous research [10]. Then, four features were selected through importance analysis. The importance analysis is the process of “feature selection” in our process. The importance of the features was calculated with different methods (Gini for random forest and gain-based method for XGBoost), and then the most important features based on the important metrics were selected to further optimize the models. The features are a mixture of PPG-based physiological characteristics, signal-directed characteristics, and physical parameters. Three additional external input features were added to determine the final seven features. These extracted features were as follows: zero-crossing rate (ZCR); autocorrelation (ACR); kurtosis (kurt), variance (var), and mean of power spectral density (PSD); kurtosis (kurt), variance (var), mean, and skewness (skew) of Kaiser–Teager energy (KTE); kurtosis (kurt) and skewness (skew) of spectral analysis (spec); mean of wavelet analysis; autoregressive coefficients (AR); skewness; sum of absolute difference (SAD); body mass index (BMI), blood oxygenation (SpO_2_); finger width (FW). Detailed explanations of these features can be found in [10].

After calculating the abovementioned features, the final feature vector was obtained using Equation (1) for each frame of the PPG signal.

(1) $$X_{F}^{f}=\left[\begin{array}{c} s_{zcr},s_{ACR},\;s_{PSD}^{kurt},\;s_{PSD}^{var},\;s_{PSD}^{mean},s_{KTE}^{kurt},s_{KTE}^{var},s_{KTE}^{mean},s_{KTE}^{skew},s_{spec}^{kurt},\;s_{spec}^{skew},\\ s_{wavelet}^{mean}\;,s_{AR}\;,s_{spo2},s_{skew},s_{SAD},BMI,\;FW \end{array}\right]$$

### 3.3. Estimation of Glycated Hemoglobin Using ML

In this study, both random forest (RF) [11] and extreme gradient boosting (XGB) [12] were used to derive the ML results. These two models exploit ensemble methods in their own algorithms. The ensemble technique has the effect of remarkably reducing overfitting, which is likely to occur in a PPG signal measurement method that utilizes the biometric information of the human body. In addition, since complex biosignals are used, it is difficult to understand the characteristics of the corresponding signals using a single and simple model. On the other hand, the ensemble method, which has been confirmed to be effective for estimating blood-glucose levels in our previous study using PPG signals [10], was selected here.

Although RF and XGB are both ensemble methods, they follow different algorithms for the final decision. For example, RF works on the principle of averaging methods. On the other hand, XGB uses a boosting method. In the RF algorithm, many random decision trees are created. All data samples are passed through these trees. At the final stage, decisions from all trees are averaged or voted to provide an output for classification or regression problems. The algorithm is also known as bagging. In contrast, XGB uses a sequence of weak models to provide a strong prediction. If we consider N weak models in the XGB classifier, the first model takes the original input and gives a weighted output. Then, the weighted output is passed through the second model as an input, and this process continues until the Nth model. At the last stage, the weighted output from the model is considered the final output. This method is also called boosting.

**Figure 3:**
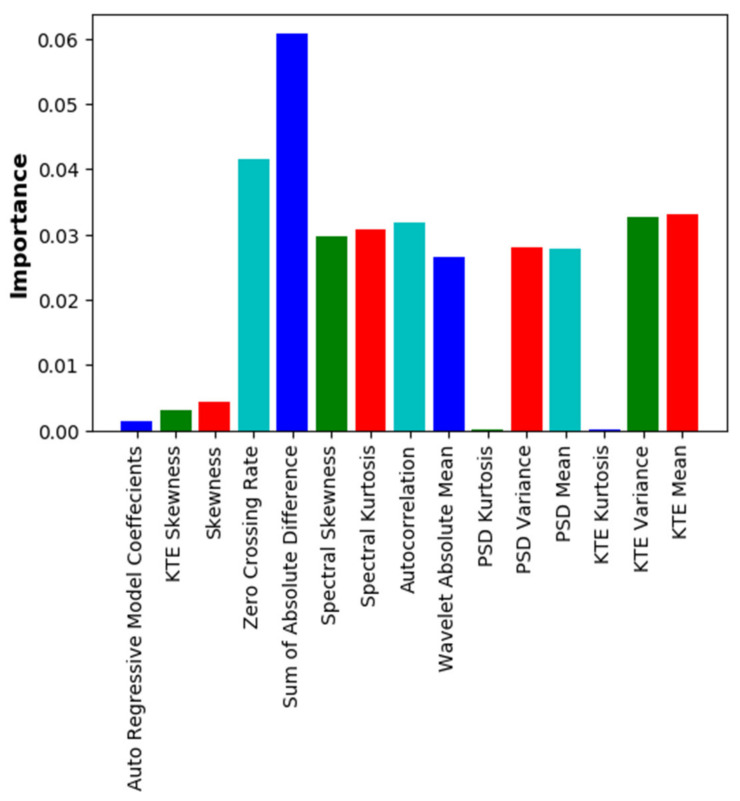
Importance of individual features in RF model (excluding BMI, SpO_2_, and FW).

**Figure 4:**
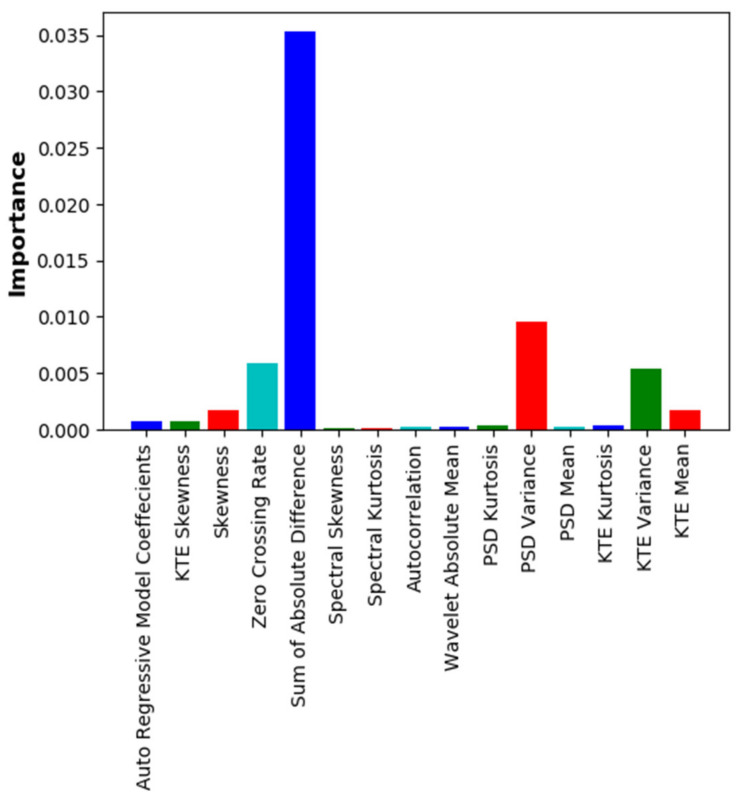
Importance of individual features in XGB model (excluding BMI, SpO_2_, and FW).

The importance of the existing features, except for the features of external information (BMI, FW, SpO_2_), was identified, and features with significantly lower importance were removed. The importance was confirmed using both the RF and XGB algorithms. Figure 3 shows the importance of each feature in the RF model for the 15 chosen features, and Figure 4 shows the importance of each feature in the XGB model. In both cases, the external input features were excluded.

**Figure 5:**
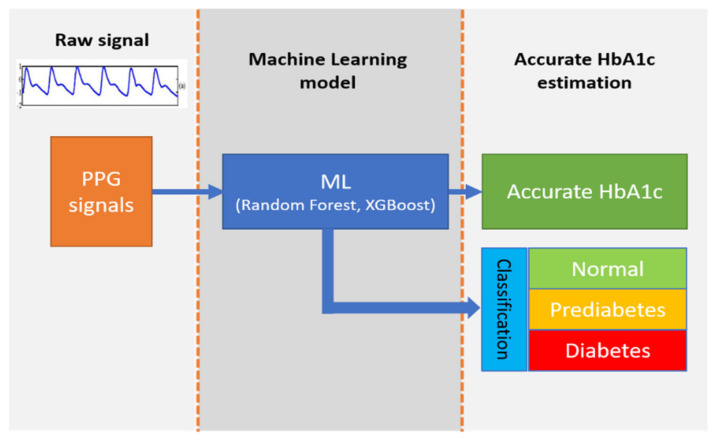
Block diagram of the proposed ML-based HbA1c measurement system.

Finally, four representative features (sum of absolute difference, PSD variance, KTE variance, and ZCR) were extracted, and learning was conducted using these four representative features and three external input features (BMI, FW, and SpO_2_), i.e., a total of seven features. In addition, by analyzing the estimated glycated hemoglobin values, subjects can be categorized into three classes (normal, prediabetes, and diabetes). In the case of glycated hemoglobin levels, subjects with values exceeding 6.5 are considered to be in the diabetes class, those with values in the range of 5.7–6.4 are considered to be in the prediabetes class, and those with values less than 5.6 are in the normal class. Figure 5 shows a block diagram of the proposed ML system.

## 4. Results and Discussion

### 4.1. Experimental Device

For the experiments, we developed a device to collect PPG signals. The hardware part developed herein consists of two independent PPG types: reflective and transmissive. However, the processor configuration was the same for both cases. We developed a SEN0212-based device that uses white-light LEDs. The wavelength selection of the LED source in the experimental setup was based on the results of our previous study [13]. The device includes reflective- and transmissive-type PPG signals, and its structure is composed of a reflective LED, a transmissive LED, and a common PD, as shown in Figure 1. The reason for dividing the LED into the reflective and transmissive types and using a common PD is that the light intensities of the reflective and transmissive type LEDs are set differently. In the case of the SEN0212 used in our study, the light intensities of the reflection and transmission type LEDs are 52 mW and 1 W, respectively.

Unlike the existing model-based methods [7,8,9], the necessity for three light sources is not absolute; hence, results can be derived with one, two, or three light sources. The wavelengths of the LEDs used are red (615 nm), green (525 nm), and blue (465 nm). We used a white-light source with three wavelengths and a PD to receive the signals at the wavelengths of choice using light source filters for each of the wavelengths. Both the reflective and transmissive systems use the same LED wavelengths and a common PD.

The microcontroller used here was an Arduino UNO. The commercial sensor module DFRobot SEN0212 consists of a color sensor (TCS34725) and a set of white LEDs. The TCS34725 model is a sensitive device with band-pass filters at three wavelengths (R, G, B) and an infrared (IR) cut filter. The sensor can be operated with a sampling rate of around 56 Hz using the proposed protocol.

**Figure 6:**
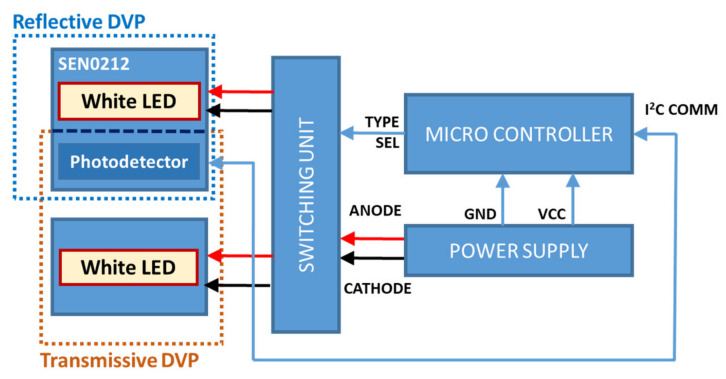
System block diagram of the SEN0212-based device [7].

For the reflective-type system, the white-light LEDs and PD are placed side by side, with the PD being used to record the reflected signals. Further, for the transmissive-type system, a separate high-power white-light LED is attached to the opposite side of the PD. The switching device supplies power to only one of the LEDs (transmissive or reflective) according to the “Type Sel” signal from the microcontroller. The “Type Sel” signal automatically changes the mode of the device (transmissive or reflective) every minute. The LED and sensor modules are packaged in the form of a clip and attached to the tip of the finger. Figure 6 shows the system block diagram of the SEN0212-based device.

### 4.2. Data Acquisition

After developing the hardware system, the PPG data were collected from subjects. A total of 4 min of reflected and transmitted PPG signals were collected from each subject. A prototype device was built and used to collect the PPG signals required for the experiments, and the data were measured and collected using a Python-based application. The application is designed to easily save the data stream sent from the hardware to the PC easily by automatically managing the volunteer IDs and data files.

**Figure 7:**
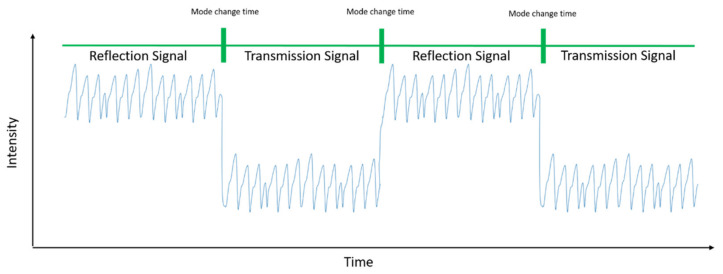
Obtained PPG signal from reflection and transmission modes of the device.

For 4 min of signal measurements, the device switches between the transmission and reflection modes at 1 min intervals, thus measuring in the reflection and transmission modes for 2 min each. This ensures that problem-free and sufficient data collection even if external factors (such as a finger movement) temporarily prevent signal reception or cause errors. Figure 7 shows the signal acquisition scenario used in this study.

Commercial products used to measure the glycated hemoglobin and SpO_2_ were used herein to collect reference data for calibration and verification. An invasive Biohermes A1c EZ HbA1c checker was used as the reference device for measuring glycated hemoglobin. A noninvasive Schiller ARGUS OXM plus was used as the reference device for measuring SpO_2_, which is additionally required information. In addition to the PPG signals, the subjects’ BMI and FWs were collected. The subjects for the study were recruited through institutional review board (IRB) deliberation (approval number: KMU-202006-HR-237) of Kookmin University, Seoul, Korea, and data were collected according to the IRB procedures. The devices used in the data collection are listed in Table 1.

**Figure 8:**
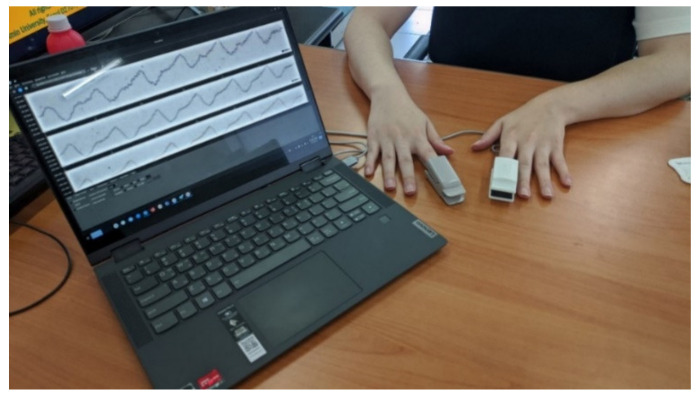
PPG signal measurement setup and acquisition experiment (a noninvasive commercial SpO_2_ device in the right hand and a PPG device developed by us in the left hand).

Figure 8 shows an image of the measurement and collection of PPG signals using the abovementioned devices. As for the ground truth, HbA1c was measured only once for each subject since it reflects the average blood glucose level over 3 months. For SpO_2_, an average of the four-minute measurements was used at a time similar to the time when HbA1c was measured. Data from a total of 40 subjects were obtained under IRB instructions (No: KMU-202006-HR-237), as shown in Table 2.

### 4.3. Experimental Results

The Clark error grid analysis (EGA) [16] was performed to confirm the performance results of the proposed noninvasive glycated hemoglobin estimation method. The entire grid was divided into five regions (A, B, C, D, and E), where “A” represents clinically accurate data, and “B” shows various data outside the range of 20% from the baseline but within allowable range without fear of wrong judgment. The region “C” represents risky data that can lead to erroneous treatment decisions. However, a good agreement could not be confirmed from a high correlation between the two methods. Therefore, as an additional performance verification method, the Bland–Altman (B&A) plot [17], which shows the correspondence between different data, was used. This method shows the agreement between two different methods for measuring the same parameter. The closer the mean of the difference between the two methods to zero the better. The B&A plot also helps to identify the outliers (outside of the ±1.96 SD line). The total number of data used for training was 40.

The learning algorithms used were RF and XGB. For setting the training environment of both the RF and XGB models, a total of 100 trees were used, and no maximum depth limitations were given. We took advantage of the leave-one-out cross-validation (LOOCV) method to evaluate the performance of our overall model. In this LOOCV method, the training and testing phases are performed in a number of iterations. The number of iterations is equal to the number of volunteers in our dataset (*n* = 40). In each iteration, one volunteer’s data were kept in the test dataset, and the other volunteers’ data were kept in the training dataset (*n* = 39). Then, from the test data, 1% of the total signal window was moved to the training dataset. In this process, a total of 40 training-test were performed by changing the testing subject.

After deriving results using the EGA and B&A plots, the performance was verified through error analysis. Since it is possible to use 1–3 different wavelengths for glycated hemoglobin estimation with ML, the performance of each wavelength combination was evaluated to find the optimal wavelength combination. Additionally, we checked the results with the addition of FW as a feature, which is an important factor. Thereafter, performance comparisons were performed methods based on our previous model. In Table 3, the Pearson correlation coefficients (Pearson’s r) are listed for all wavelength combinations.

From the performance results of each wavelength combination for both RF and XGB algorithms, the case with all three (RGB) wavelengths was observed to be the best. However, this has the disadvantage that the maximum wavelength combination (three wavelengths) is used. Notably, the reflection-type XGB algorithm showed relatively higher performance, and both RGB and RG combinations had the same Pearson’s r values. This is consistent with the results of a previous study indicating that there are differences in the absorption of oxyhemoglobin and deoxyhemoglobin at each wavelength and that the RG combination is the best among two-wavelength combinations [18]. Hence, the RG wavelength combination was finally selected. Overall, by considering both performance and the number of wavelengths required, the RG combination was selected as the most efficient choice.

Performance comparisons were also performed when a total of 18 features were used, by adding the FW feature to the 17 features used in a previous blood-glucose estimation study [10], and when only 7 features were used after excluding unnecessary features. Table 4 shows the performance comparison for the cases using 18 and 7 features after excluding low-importance features. The case with 7 features showed better performance than that when all 18 features were used; the reason for this is that some of the existing features with significantly lower importance values may actually cause learning errors. In fact, from checking the results of feature importance analysis (Figure 3 and Figure 4), it was observed that some features had remarkably low importance values.

**Figure 9:**
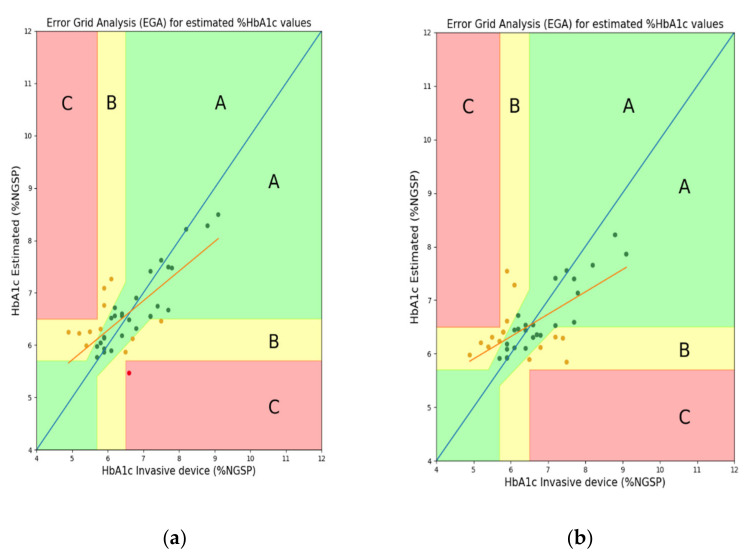

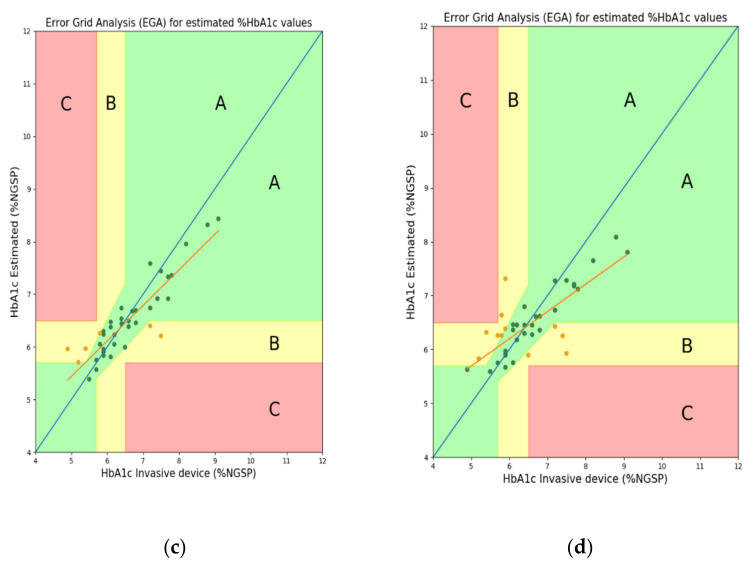
EGA plots when finger width was not used as a feature: (**a**) RF reflection, (**b**) RF transmission, (**c**) XGB reflection, and (**d**) XGB transmission systems.

**Figure 10:**
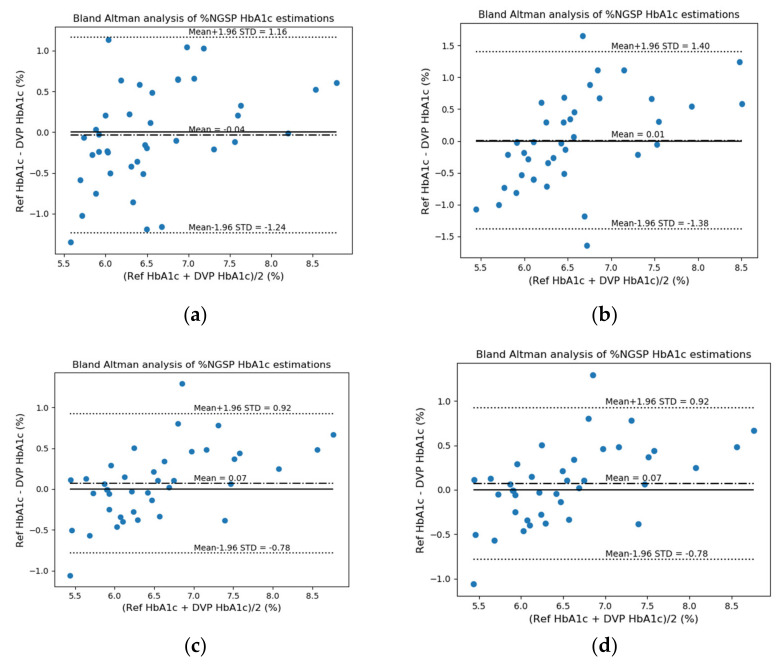
B&A plots when finger width was not used as a feature: (**a**) RF reflection, (**b**) RF transmission, (**c**) XGB reflection, and (**d**) XGB transmission systems.

**Figure 11:**
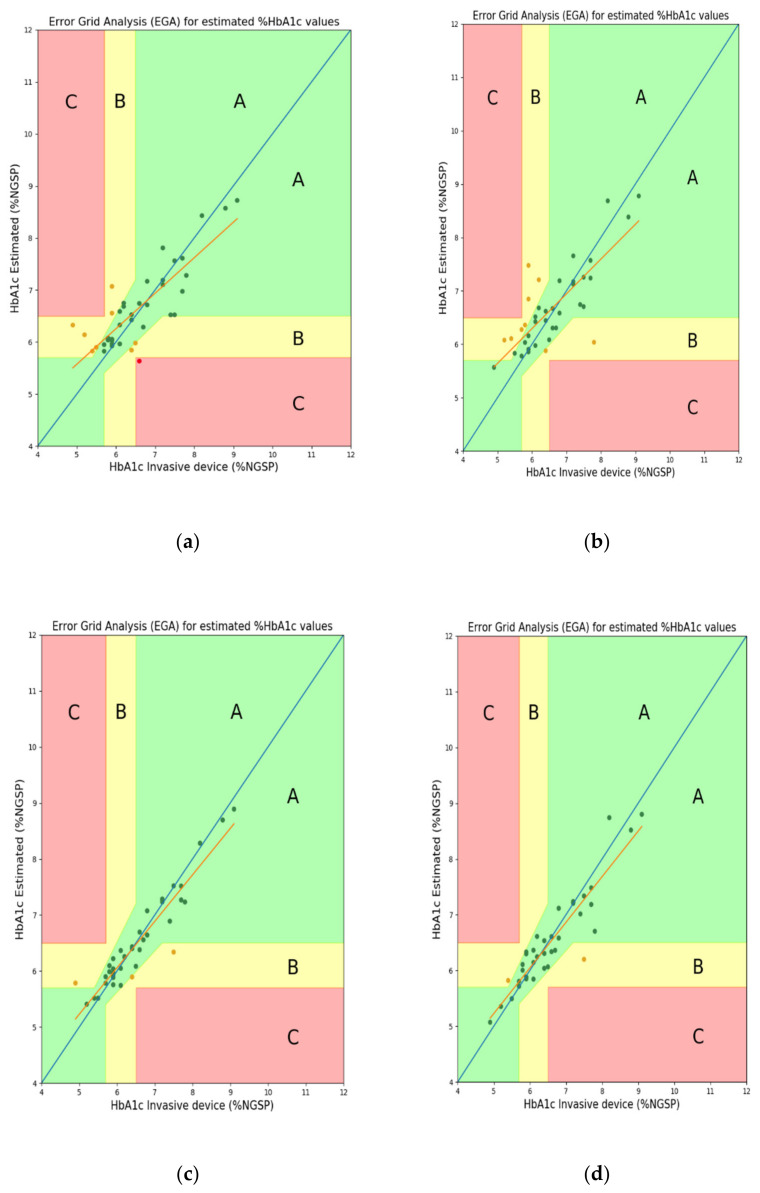
EGA plots when using finger width as a feature: (**a**) RF reflection, (**b**) RF transmission, (**c**) XGB reflection, and (**d**) XGB transmission systems.

**Figure 12:**
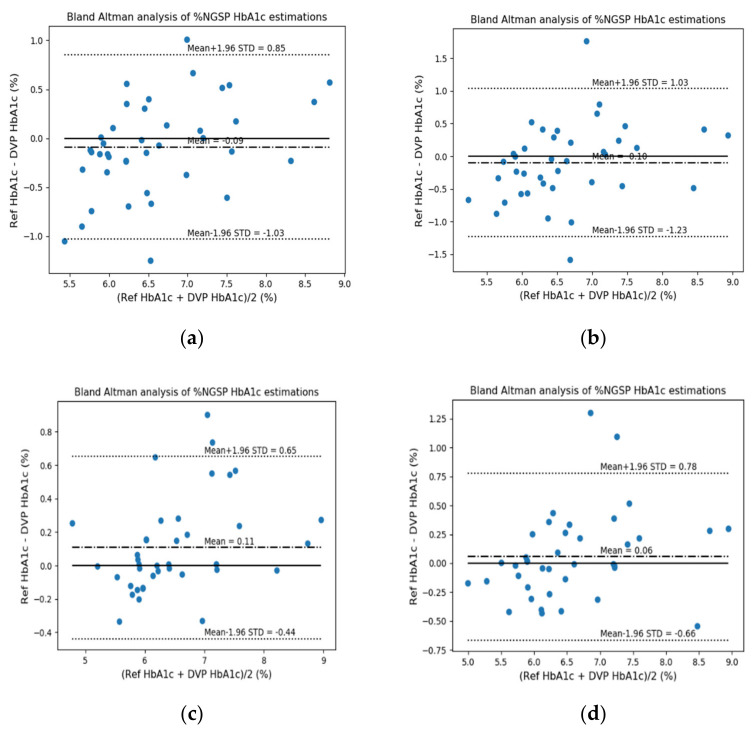
B&A plots when using finger width as a feature: (**a**) RF reflection, (**b**) RF transmission, (**c**) XGB reflection, and (**d**) XGB transmission systems.

For error analysis, the differences in standard deviation (Diff STD), mean-squared error (MSE), mean error (ME), mean absolute deviation (MAD), root-mean-squared error (RMSE), *R*^2^ score, and Pearson’s r were used. Then, we confirmed the performance differences based on whether the FW was used as one of the features. The FW feature has not been used in the previous study [10]; however, this feature is an important parameter for glycated hemoglobin estimation and may also be an important parameter for estimating blood glucose levels. FW can be useful for predicting the degrees of absorption and reflection of the wavelengths (light) irradiated onto the finger in both transmission- and reflection-type systems. Figure 9 and Figure 10 show the EGA and B&A plots, respectively, when the FW was not used as a feature. Figure 11 and Figure 12 show the EGA and B&A plots, respectively, when the FW was used as a feature.

The importance of FW as a feature can be visualized in Figure 9, Figure 10, Figure 11 and Figure 12. In Figure 9, we observe that the fitted line deviates considerably from the ideal linear line and that a good number of predicted data points are in region “B”. In the B&A plot analysis for the same case, we can again see that the mean difference between data from the proposed prediction model and reference device data is larger and that is why most of the data fall outside the mean line. Nevertheless, using FW in the feature set, we find the opposite scenario, which is depicted in Figure 11 and Figure 12.

From the error analysis results with and without FW as a feature, it was confirmed that the performance can be greatly improved when FW is used. This is because FW determines the distance between the transmitter and receiver, thereby affecting both the transmission- and reflection-type systems. Thus, the FW can be useful information for predicting the degrees of absorption and reflection of the wavelengths (light) irradiated on the finger. In addition, the finger-clip-type device works based on the pressure between the finger and sensor according to the finger thickness. The thicker the finger, the greater the pressure. Therefore, FW causes a difference in the pressure applied to the sensor, which significantly affects the signal; hence, it was confirmed that the performance is improved when the FW information is used as a feature.

Table 5 shows the error analysis results with and without using the FW feature. In this analysis, we again confirmed the importance of FW through quantitative measurements. An XGB regressor with the FW feature works best except for the “mean error (ME)” metric. From the error analysis results with and without FW as a feature, it was confirmed that the performance can be greatly improved when using the FW data. The reason for this is that FW determines the distance between the transmitter and receiver, thereby affecting both the transmission- and reflection-type systems. Thus, the FW can be useful information for predicting the degrees of absorption and reflection of the wavelengths (light) irradiated on the finger. In addition, the finger-clip-type device works based on the pressure between the finger and the sensor according to the finger thickness. The thicker the finger, the greater the pressure. Therefore, the FW causes a difference in the pressure applied to the sensor, which has significant effects on the signal; hence, it was confirmed that the performance is improved when the FW information is used as a feature.

The metrics for examining the performance of the models are the standard deviation of the difference (Diff STD), mean-squared error (MSE), mean error (ME), mean absolute deviation (MAD), root-mean-squared error (RMSE), *R*^2^ score, and Pearson’s r. The equation for the standard deviation of the difference is given below.

(2) $$Diff\;STD=standard\;deviation\;\left(reference-estimated\right)$$

From the experimental results, the proposed ML-based glycated hemoglobin estimation method uses both red and green wavelengths and shows the best performance when using ML (XGB) with seven features, including FW. Table 6 shows the performance comparison of the existing model-based and proposed ML-based glycated hemoglobin estimations. In this table, we can observe the superior performance of the proposed ML-based method. The existing computational models used for comparison rely on the modeling of the human blood components and finger structure. However, for the sake of simplicity, many complex phenomena are omitted from these models. On the other hand, machine learning methods can find those complex relationships between input features and targets. 

**Figure 13:**
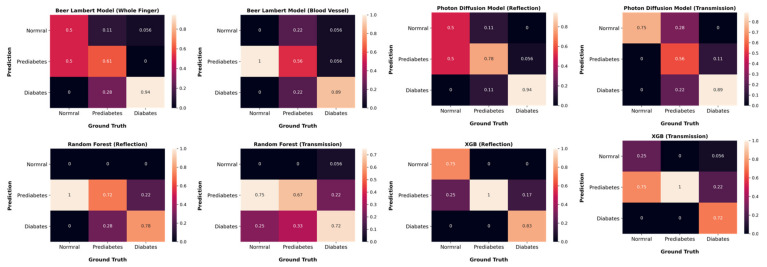
Confusion matrix for diagnosing diabetes between the proposed ML-based (using R and G wavelengths and 7 selected features) and our previous model-based methods.

To evaluate the classification accuracy, the diabetes status diagnosis performance was confirmed by comparing the glycated hemoglobin estimated values from the proposed method with reference values. Table 7 shows the performance comparisons for diagnosing diabetes status, respectively, between the proposed ML-based and our previous model-based methods. The proposed XGBoost (reflection) method shows the best prediction performance; in particular, the most dangerous false-negative area (red area) in the diagnosis had the least results. Thus, the superiority of the proposed method was proved. Figure 13 and Table 7 show the confusion matrix and performance comparison for diagnosing diabetes status, respectively, between the proposed ML-based method and our previous model-based methods. In Figure 13, we can see that all models perform well to classify patients as diabetic, but the photon-diffusion-based reflection model shows the best performance. Prediabetes is also successfully classified; for this classification, Beer–Lambert-based blood vessel model and photon-diffusion (transmission) model do not perform well. It is worth noting that, overall, the proposed XGBoost (reflection) method shows relatively superior prediction performance. Thus, the superiority of the proposed method was proven.

Finally, it was confirmed that the proposed ML-based glycated hemoglobin estimation method shows effective performance for diagnosing diabetes status. From Table 7, we can see that XGB performs better than other models in terms of diabetes classification accuracy. PPG signal for the red and green light and all features except BMI and SpO_2_ combination were used, obtaining 90% accuracy. On the other hand, photon diffusion theory shows the best performance among the previous model-based methods. Thus, the RG (red and green) wavelength combination and seven features including FW were selected for our XGB reflection model.

## 5. Discussion

In this study, we developed an end-to-end methodology to measure HbA1c using machine learning models. First, we developed a hardware design and circuit consisting of two white LEDs: one for reflection and the other for transmission. The PD used can separate the red, green, and blue light from the incident light. A compact PC software was also been developed to visualize and store data from our PPG device. In the second step, we suggested some vital features based on the signal properties and body parameters to provide as inputs to the ML models. Two popular ensemble methods were explored: random forest (RF) and extreme gradient boosting (XGB). We conducted experiments on feature importance and found that seven features are the most important. In addition, we established finger width (FW) as a vital feature, and we observed a decline in the performance level of the model without the FW feature. Besides this observation, more investigation was carried out on the combination of LEDs, and the red–green (RG) pair was the most successful. We compared our results against some previous computational models based on the Beer–Lambert law and photon diffusion theory. Our proposed method outperformed those models in terms of regression analysis and classification accuracy. Overall, XGB showed better performance than random forest. There might be two possible reasons. XGB performs better than random forest in the case of unbalanced datasets. The random forest algorithm prioritizes hyperparameters more, while the XGB algorithm focuses on functional space to reduce the model cost. Although our models performed better than other comparative methods, this study also faced some drawbacks. It should be noted that the use of BMI and FW limits the generalization of this method because these have to be input externally. Therefore, efforts will be made in the future to capture this property from the acquired PPG signal. This study would have a greater impact if a more diverse and balanced dataset can be collected. In many cases, poor performance can occur due to an imbalanced dataset. Hence, in the future, we will focus on expanding the dataset. Moreover, we also plan to explore this domain using deep learning.

## 6. Conclusions

Glycated hemoglobin is an essential element for identifying and managing diabetes in subjects. However, all of the currently used measurement devices are invasive and require blood extraction; although some studies have attempted methods to measure glycated hemoglobin noninvasively in the past, there are few cases where optical signals are used for practical purposes. There are no practical means to guarantee the performance of noninvasive estimations. In the present study, a noninvasive method and apparatus for estimating HbA1c were proposed using machine learning methods. PPG signals were used as noninvasive input signals, and the optimal wavelength combination for estimating glycated hemoglobin with 1–3 different wavelengths (R, G, and B) was selected via experiments. In addition, 18 features for glycated hemoglobin estimation were extracted, and 7 features with the best performance were selected through feature importance analysis; two methods (RF, XGB) of machine learning were also investigated using these features. The practical applicability of noninvasive glycated hemoglobin estimation was confirmed with a prototype-embedded device using the proposed method. Furthermore, the superiority of the proposed ML method was demonstrated through performance comparisons of error analysis and diabetes status diagnosis with the existing model-based models—namely, models based on the Beer–Lambert Law [7] and photon diffusion theory [8]. This approach can be considered a stepping stone for further research in this field. In the near future, in cooperation with medical centers, we intend to improve the performance of the proposed method by acquiring additional clinical data and plan to improve and expand the proposed method through deep learning.

## Figures and Tables

**Table 1:** Data acquisition devices and products.

Device	Picture	Purpose
SEN0212-based device [10]	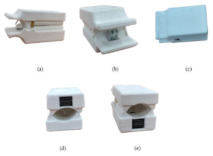	PPG signal measurement (R, G)
Schiller ARGUS OXM plus [14]	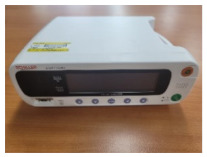	SpO_2_ measurement for reference
Biohermes A1c EZ HbA1c checker [15]	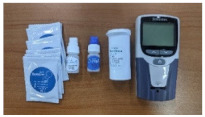	HbA1c measurement for reference

**Table 2:** Data obtained from the experiments.

Subject No.	BMI	FW (cm)	SpO_2_ (%)	HbA1c (%)
1	23.4	1.2	97	6.7
2	20	1.2	96	6.6
3	31.1	1.5	94	7.2
4	25.7	1.1	97	7.8
5	31.1	1.3	95	6.4
6	27.3	1.2	97	7.5
7	27.7	1.5	97	6.8
8	25.4	1.5	97	7.2
9	23.9	1.5	97	7.2
10	20.8	1.3	97	6.1
11	25.7	1.6	98	6.2
12	27.7	1.2	99	6.5
13	20.8	1.1	96	6.4
14	20.8	1.4	97	6.6
15	25.4	1.3	94	7.5
16	31.1	1.2	96	7.4
17	25.4	1.3	96	7.7
18	22.1	1.5	98	7.7
19	21.5	1.2	96	6.8
20	30.7	1.3	97	5.9
21	28.8	1.1	97	4.9
22	31.9	1.3	96	5.7
23	33.1	1.4	96	5.9
24	27	1.4	99	5.2
25	25.3	1.3	97	6.2
26	32.2	1.4	97	5.9
27	31.9	1.3	95	5.7
28	21.6	1.3	97	5.8
29	33.1	1.5	95	8.8
30	33.1	1.5	96	8.2
31	25.9	1.1	97	5.9
32	27.6	1.2	98	5.4
33	27.8	1.3	95	5.8
34	33.1	1.5	98	9.1
35	31.9	1.3	97	5.9
36	23.5	1.1	96	6.4
37	24.4	1.2	98	6.1
38	28.1	1.3	97	6.1
39	24	1.1	93	5.5
40	32.9	1.3	97	5.9

**Table 3:** Pearson’s r according to wavelength combination with 7 features.

Wavelength Combination	Random Forest		XGB	
	Reflection	Transmission	Reflection	Transmission
R	0.8	0.63	0.9	0.82
G	0.83	0.81	0.91	0.93
B	0.81	0.78	0.89	0.89
RG	0.86	0.79	**0.96**	0.92
RB	0.79	0.80	0.93	0.91
GB	0.80	0.80	0.93	0.86
RGB	**0.94**	**0.91**	**0.96**	**0.94**

Best values are in boldface font. R: Red, G: green, B: blue, RG: red–green, RB: red–blue, GB: green–blue, RGB: red–green–blue.

**Table 4:** Performance comparison between 18 features and 7 features for RG wavelength combination.

	18 Features				7 Features			
	Random Forest		XGBoost		Random Forest		XGBoost	
	Reflection	Transmission	Reflection	Transmission	Reflection	Transmission	Reflection	Transmission
Diff STD	0.523	0.675	0.321	0.439	0.481	0.576	**0.278**	0.368
MSE	0.279	0.467	0.105	0.195	0.239	0.341	**0.089**	0.139
ME	−0.075	−0.106	**0.039**	0.051	−0.088	−0.096	0.108	0.058
MAD	0.404	0.483	0.220	0.276	0.380	0.440	**0.201**	0.260
RMSE	0.529	0.683	0.324	0.442	0.489	0.584	**0.298**	0.373
$R^{2}$ score	0.685	0.473	0.881	0.780	0.730	0.615	**0.900**	0.843
Pearson r	0.831	0.709	0.942	0.884	0.862	0.791	**0.957**	0.921

Best values are in boldface font.

**Table 5:** Error analysis results on whether or not finger width is applied.

	w/o FW				with FW			
	Random Forest		XGBoost		Random Forest		XGBoost	
	R	T	R	T	R	T	R	T
Diff STD	0.612	0.709	0.435	0.601	0.481	0.576	**0.278**	0.368
MSE	0.376	0.503	0.194	0.368	0.239	0.341	**0.089**	0.139
ME	−0.037	**0.008**	0.071	0.082	−0.088	−0.096	0.108	0.058
MAD	0.493	0.567	0.335	0.465	0.380	0.440	**0.201**	0.260
RMSE	0.613	0.710	0.441	0.607	0.489	0.584	**0.298**	0.373
$R^{2}$ score	0.575	0.432	0.780	0.584	0.730	0.615	**0.900**	0.843
Pearson’s r	0.760	0.657	0.899	0.781	0.862	0.791	**0.957**	0.921

Best values are in boldface font.

**Table 6:** Performance comparison of the proposed machine-learning-based method with our previous model-based methods.

	Beer-Lambert [7]		Photon Diffusion [8]		Proposed (RF)		Proposed (XGB)	
	Blood Vessel	Whole Finger	Reflection	Transmission	Reflection	Transmission	Reflection	Transmission
Diff STD	0.581	0.731	0.508	0.568	0.481	0.576	**0.278**	0.368
MSE	0.338	0.590	0.259	0.328	0.239	0.341	**0.089**	0.139
ME	**−0.020**	−0.237	−0.022	0.077	−0.088	−0.096	0.108	0.058
MAD	0.461	0.578	0.385	0.462	0.380	0.440	**0.201**	0.260
RMSE	0.581	0.768	0.509	0.573	0.489	0.584	**0.298**	0.373
$R^{2}$ score	0.619	0.334	0.708	0.629	0.730	0.615	**0.900**	0.843
Pearson r	0.805	0.745	0.862	0.818	0.862	0.791	**0.957**	0.921

Best values are in boldface font.

**Table 7:** Comparison of diabetes diagnosis performance between the proposed ML-based and our previous model-based methods.

	Beer–Lambert Model		Photon-Diffusion Model		Random Forest		XGBoost	
	Blood Vessel	Whole Finger	R	T	R	T	R	T
Accuracy	65%	75%	82.5%	72.5%	67.5%	62.5%	**90.0%**	80%
Precision	0.48	0.68	0.74	0.81	0.5	0.46	**0.86**	0.66
Recall	0.49	0.67	0.74	0.67	0.45	0.43	**0.94**	0.74
F1 score	0.48	0.67	0.74	0.73	0.47	0.44	**0.90**	0.70

Best values are in boldface font.

## Data Availability

The dataset used in this research is available upon a valid request to any of the authors of this research paper.

## Declaration

This statement is to certify that all Authors have seen and approved the manuscript being submitted. We warrant that the article is the Authors’ original work and has not received prior publication and is not under consideration for publication elsewhere.
